# Deciphering colorectal cancer immune microenvironment transcriptional landscape on single cell resolution – A role for immunotherapy

**DOI:** 10.3389/fimmu.2022.959705

**Published:** 2022-08-10

**Authors:** Francis Yew Fu Tieng, Learn-Han Lee, Nurul-Syakima Ab Mutalib

**Affiliations:** ^1^ Universiti Kebangsaan Malaysia (UKM) Medical Molecular Biology Institute (UMBI), Universiti Kebangsaan Malaysia, Kuala Lumpur, Malaysia; ^2^ Novel Bacteria and Drug Discovery Research Group, Microbiome and Bioresource Research Strength, Jeffrey Cheah School of Medicine and Health Sciences, Monash University Malaysia, Subang Jaya, Selangor, Malaysia; ^3^ Faculty of Health Sciences, Universiti Kebangsaan Malaysia, Kuala Lumpur, Malaysia

**Keywords:** immune landscape, single cell, colorectal cancer, metastasis, tumor immune microenvironment, immunotherapy, precision medicine

## Abstract

Single cell RNA sequencing (scRNA-seq) is a novel high-throughput technique that enables the investigation of a single cell’s entire transcriptome. It elucidates intricate cellular networks and generates indices that will eventually enable the development of more targeted and personalized medications. The importance of scRNA-seq has been highlighted in complex biological systems such as cancer and the immune system, which exhibit significant cellular heterogeneity. Colorectal cancer (CRC) is the third most common type of cancer and the second leading cause of cancer-related death globally. Chemotherapy continues to be used to treat these patients. However, 5-FU has been utilized in chemotherapy regimens with oxaliplatin and irinotecan since the 1960s and is still used today. Additionally, chemotherapy-resistant metastatic CRCs with poor prognoses have been treated with immunotherapy employing monoclonal antibodies, immune checkpoint inhibitors, adoptive cell therapy and cancer vaccines. Personalized immunotherapy employing tumor-specific neoantigens allows for treating each patient as a distinct group. Sequencing and multi-omics approaches have helped us identify patients more precisely in the last decade. The introduction of modern methods and neoantigen-based immunotherapy may usher in a new era in treating CRC. The unmet goal is to better understand the cellular and molecular mechanisms that contribute to CRC pathogenesis and resistance to treatment, identify novel therapeutic targets, and make more stratified and informed treatment decisions using single cell approaches. This review summarizes current scRNA-seq utilization in CRC research, examining its potential utility in the development of precision immunotherapy for CRC.

## Introduction

According to the International Agency for Research on Cancer, colorectal cancer (CRC) was the third most commonly diagnosed cancer and ranked second in global cancer-related mortality in 2020 (1). Approximately 20% of the newly diagnosed CRC patients developed distant metastases, while another 25% with localized tumors will eventually acquire metastases (2, 3). The expected 5-year survival decreases from 82%-90% in stage I or II CRC patients to a dismal 12% in those of stage IV (4). Currently, surgery alone is still the gold standard for stage I and II cancer, while for some stage II, III and early-stage IV CRC patients with minimal metastases, neoadjuvant, or adjuvant chemotherapy coupled with surgery is still an option (4). Although these conventional cancer therapies target the tumor cells and yield positive outcomes in the majority of CRC patients, the benefits are often offset by tumor reoccurrence and chemoresistance after prolonged treatment (5). In short, metastasis is still a major challenge in CRC’s effective treatment (6).

Conversely, immunotherapy exploits the immune system to induce a systemic response targeting malignant tumor cells (7–9). The discovery of first-generation antibody-based immunotherapy, also known as immune checkpoint inhibitor (ICI), altered the CRC therapeutic landscape. It functions *via* preventing anti-programmed cell death ligand 1 (PD-L1) and anti-cytotoxic T lymphocyte-associated antigen-4 (CTLA-4) monoclonal antibodies (mAbs) from binding/interacting with the receptors during T cell activation, allowing the latter to recognize and destroy CRC cells (10, 11). Nivolumab (Opdivo^®^; PD-1 blocker) is the most extensively studied ICI in metastatic CRC (mCRC) (12–14). Additionally, combinational administration with Ipilimumab (Yervoy^®^; CTLA-4 blocker) has also shown improvement in overall survival and long-term treatment responses particularly in mCRC patients with microsatellite instability-high (MSI-H) and mismatch repaired (MMR) aberrations (15–17). Nevertheless, the success of CRC immunotherapy remains unsatisfactory since positive responses are confined to a minority of patients (18, 19).

Successful tumor control by immunotherapy is observed only in metastatic CRC (mCRC) of MMR and MSI-H with immune infiltration, indicating that CRC and its treatment response could be influenced by the tumor immune microenvironment (TIME) (20, 21). Interplay underlying immunotherapy and the TIME is therefore vital not only for deciphering the mechanisms of action but also for identifying advanced biomarkers and revising current immunotherapy strategies for better efficiency (22, 23). In this regard, single cell technology has quickly emerged as a potent technique to investigate the TIME in mCRC. Single cell RNA-sequencing (scRNA-seq) not only provides an unprecedented, detailed characterization of transcriptomes of cell diversity and heterogeneity in immune cell populations but also potentially discovers novel cellular or molecular factors involved in ICI, thereby allowing comprehensive assessment of the complexity of TIME (24, 25). Henceforth, scRNA-seq will be firmly embedded as a tool in oncology, with increasing incorporation of genes/neoantigens into the cancer panels, and the fusion of immunotherapy with scRNA-seq is expected to deliver truly precision treatment to an expanding number of mCRC patients.

## Tumour immune microenvironment reshapes the current transcriptional landscape

**Figure 1:**
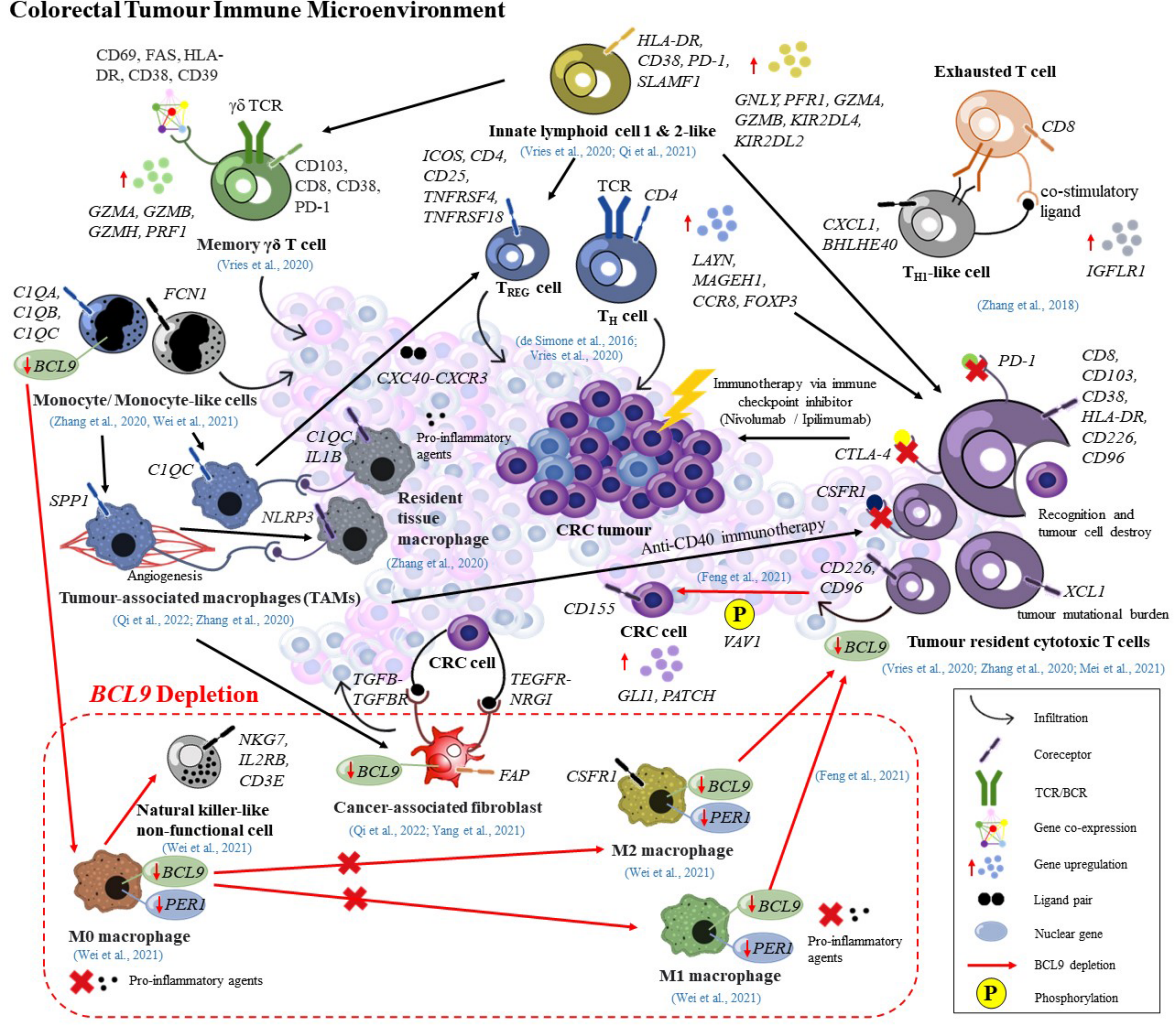
Potential immune-based precision medicine targets governed by scRNA-seq.

Recent advances in cancer immunotherapy encounter a bottleneck due to the complex tumor microenvironment (TME) which provides a formidable barrier to immune infiltration and function (26–28). The TME consists of various cell types including transformed cancer cells from the epithelium of the tissue of origin; stromal cells/non-cancer cells (fibroblasts, adipocytes, endothelial, immune cells) and the extracellular matrix. As the heart of TME, cancer cells exploited non-malignant cells to prevent recognition and elimination by T cells, followed by establishing dormant tumor immune tolerance *via* ‘immunoediting’ their immunogenicity or antigen presentation as well as secreting favorable cytokines to exhaust T cells (29–31). Other consequences of such crosstalk are reflected in tumor growth maintenance, metastasis formation, deficient immunotherapeutic response and multi-drug resistance (32–35). Although single cell transcriptome analyses have been extensively used to study the relationship between TME and immunotherapy response, it remains unclear how cancer cells and host tissues differentially influence the immune composition within TME (36–38). Moreover, there is a lack of effective predictive biomarkers, making it difficult to accurately grasp the effect of immunotherapy (39, 40). As such, the transition towards tumor immune microenvironmental (TIME) transcriptional landscaping is rational given that a successful tumor control induced by immunotherapy requires the activation of the immune system, expansion of the effector cells, infiltration of activated effector cells to the tumor tissue, and elimination of the tumor cells (41). A deeper understanding of the crosstalk between cancer and immune cells not only permits dissection of the immune complexity of TME in CRC but also provides a comprehensive characterization of all immune cells that participate in modulation of TIME. Additionally, scRNA-seq studies on the immune landscape of CRC might unveil the underlying mechanisms modulating immune cells exhaustion, and the identification of advanced biomarkers, enabling the devising of novel personalized immunotherapy strategies. In the future, it is possible that single cell studies on the TIME could provide a snapshot of CRC tumor evolution since tumor cells interact with immune cells most frequently, tumor immunology and evolution are interwoven, and co-evolution has been proven to exist between them (42, 43). Thus, in this review, we aimed to summarize the current scRNA-seq studies on TIME of CRC and assess their potential utilities as immune-based therapeutic biomarkers in personalized immunotherapy illustrated in **Figure 1**.

## Fundamental importance of T lymphocytes in TIME and immunotherapy responses

The colorectal tumor immune microenvironment (TIME) is a heterogeneous microenvironment containing a variety of immune cells and their products in tumor tissues (44). Many scRNA-seq studies have found a link between significant infiltration of *CD8* ^+^ T cells (45), *CD4* ^+^ T helper 1 (Th1) (46), regulatory T (Treg) cells (47), tumor-infiltrating exhausted T cells (48), *CD45* ^+^ macrophages, dendritic cells, and myeloid cells (49), and a favourable outcome in mCRC. However, these immune cells, particularly T cells are always in a hyporesponsive state, a phenomenon often referred to as exhaustion (30). Since immunotherapy primarily relies on T cells to attack and kill tumor cells, the response of the former could be impaired if the latter’s capabilities fade, resulting in tumor immune escape, whereby CRC cells evade recognition and are not attacked by the human immune system (31, 50). Furthermore, a recent study by Sorrentino et al. has reported that exhaustion is not exclusive to T cells, but could also affect other immune cells including B lymphocytes and conventional natural killer (NK) cells from CRC patients (51). Restoring exhausted immune cells is currently an inspiring CRC therapeutic technique that is anticipated to yield promising results and mark a significant breakthrough in CRC immunotherapy. Hence, the rationale behind the shift of traditional scRNA-seq from targeting only tumor cells to neighboring infiltrated immune cells is understandable and is believed to reshape the current transcriptional landscape, redefine CRC classification, and shed insights on the tumor progression, restoration of immune cells exhaustion and immunotherapy response in CRC patients (31, 52).

The first scRNA-seq based immune transcriptional investigation was performed on CRC-infiltrating *CD4* ^+^ T cells, where the authors confirmed the influence of TIME on specific gene expressions (*LAYN*, *MAGEH1*, and *CCR8*) in tumor-infiltrating Treg cells and their correlations with immunotherapy response, tumor suppressive activity and prognosis (53). Another attempt was also performed on T cells from primary human CRC, where cell heterogeneity was discovered, and the dynamic relationships between *CD4* ^+^ and *CD8* ^+^ T cell subpopulations were explained *via* integrated transcriptomic analysis and T cell receptor (TCR) (54). Concisely, TIME plays a vital role in reshaping CRC therapeutic landscape, and high throughput technologies permit the dissection of heterogeneity of immune cells at single cell resolution as well as cell-cell interaction. Nevertheless, a comprehensive understanding of the microenvironmental interactions between tumor cells and their surrounding immune cells is still lacking since both studies focus on T cell subpopulations only.

Following this, unbiased characterization of the immune contexture of CRC was performed *via* the inclusion of all *CD45* ^+^ cells from both MMR-deficient and MMR-proficient tumors. This work explained the intricate immune landscape of primary CRC and healthy mucosa, including the discovery of a previously unappreciated innate lymphoid cells (ILCs) subpopulation and reveal a potential multitargeted immunotherapeutic response *via* modulating adaptive (cytotoxic and helper) and innate (γδ) T cells (55). In the same year, Lee and their co-workers further explored the TIME, including six immune cells (epithelial, stromal, myeloid, T, B, and mast cells) and their matched normal mucosa, followed by an illustration of an immune transcriptional landscape and reconstruction of putative interaction network between tumor cells and their surrounding microenvironment *via* the dominance of IgA-type humoral immunity in normal mucosa and γδ T-cell-driven innate immunity in CRC (56). In 2021, a significant link between B-cell and myeloid-cell signalling was revealed using two scRNA-seq approaches, while *CCL8* ^+^ cycling B cells/*CCR5* ^+^ T-cell interactions were identified as a potential antitumoral mechanism in advanced CRC tumors (57). In essence, these studies proved that T, B, and myeloid cells play a dominant role in cancer-associated immune surveillance. Profiling TIME in both healthy and neoplastic states allows uncovering of the underlying mechanisms as well as the identification of new therapeutic targets in CRC (58).

## Tumour-associated macrophage is the second significant key component after T cells

Tumour-associated macrophages (TAMs) are critical in the establishment of the TIME through their production of cytokines and chemokines, representing the most abundant immune cell population infiltrating colorectal tumors (59, 60). A detrimental effect on cancer treatment is more likely with TAMs than a beneficial effect since they are known to promote tumor angiogenesis, growth, metastasis (via miRNA-containing exosome secretion, matrix metalloproteinase 9 expression, epithelial-to-mesenchymal transition) and immunosuppression (61, 62). In conjunction with prior research findings, Chinese researchers verified in 2020 that depletion of specific infiltrating-TAMs impacted CRC immunotherapy outcomes. Based on combined scRNA-seq methods, they tracked two distinct human *C1QC* ^+^ and *SPP1* ^+^ TAM subpopulations, the latter of which exhibited inflammatory and angiogenic characteristics in human CRC and distant liver metastatic site. Moreover, they highlighted two murine TAM subsets that resembled human *SPP1* ^+^ TAM, showed resistance to anti-CSF1R depletion, and described a previously unrecognized immunological mechanism upon anti-CD40 treatment. Interestingly, they were unable to explain the dichotomy of *C1QC* ^+^ and *SPP1* ^+^ TAMs in the CRC TIME using genes related to M1- and M2-TAMs (49). In line with this, Qi et al. reported a dramatic increase in infiltrating *SPP1^+^* macrophages in CRC tissue and a positive correlation with *FAP^+^* fibroblasts, which impaired immunotherapeutic effect (63). Similarly, an expanded *SPP1* ^+^ TAM subpopulation was recovered and proposed to have both pro- and anti-inflammatory signatures *via* scRNA-seq investigation (56). Mei and her colleagues also discovered polyfunctional *SPP1* ^+^ TAM subsets in CRC that do not fit the M1 and M2 polarization paradigm (37).

Conversely, another study conducted by Hicks and her co-authors revealed that a combination of tumor-targeted interleukin-12 (*IL12*) and Entinostat therapy was capable of TAM reprogramming, resulting in a significant shift in M1/M2 TAMs balance favoring tumor resolution. Furthermore, polarization to M1-like TAMs was shown to be substantially linked with complete tumor eradication (41.7%-100%), triggered by combination therapy, correlating to antitumor efficacy (64). More recently, China experts conducted a single-cell and spatial transcriptomics analysis and identified highly metabolically active *MRC1* ^+^ *CCL18* ^+^ M2-like TAMs in colorectal liver metastasis site. They discovered that M2-like TAMs had increased metabolic activity, which might be reduced by effective neoadjuvant chemotherapy, implying the possibility of targeting metastatic metabolism pathways *via* TAM reprogramming (65). Intriguingly, Wei et al. believed that re-educating TAMs as M1 phenotype might be an efficient anticancer strategy since pro-tumor M0- and M1-TAMs were actively involved in CRC inflammation (60). Collectively, these findings indicated that targeting certain TAM subsets in conjunction with reprogramming could be beneficial in CRC treatment and immunotherapy response prediction. Unfortunately, knowledge of TAM cellular architecture and transcriptional profiles in the CRC TIME landscape remains limited.

## Remodelling of TIME *via* nuclear B-cell lymphoma 9 expression in Wnt pathway

Apart from incorporation of a wider subsets of tumor-neighboring immune cell populations to provide a more comprehensive exploration of the underlying cellular interactions during tumor progression and immunotherapy treatment, the remodelled immune translational landscape could elucidate the role of diverse signalling pathways involved in the modulation of CRC TIME. Currently, wingless-related integration site-beta catenin (Wnt/β-catenin) signalling is the best-studied pathway, whereby B-cell lymphoma 9 (*BCL9*) is the critical transcription co-factor (66). For instance, Yang et al. detected that knock down of nuclear *BCL9* promoted tumorigenesis in murine cancer-associated fibroblasts (CAFs), whereas aberrant inactivation of Wnt/β-catenin due to *BCL9* suppression aided T-cell–mediated antitumor immune responses. Briefly, the authors illustrated cellular landscape and transcription differences in CAFs upon *BCL9* depletion, as well as the reconfiguration of CRC immune surveillance in TIME *via* Wnt signalling blockage (67).

In agreement with the previous research finding, *BCL9* depletion was reported to benefit *CD8* ^+^ T cells infiltration into CRC tumor and improve anti-PD-1 immunotherapy response in murine models *via* increased *VAV1* phosphorylation in *CD8* ^+^ T cells and enhanced *GLI1* and *PATCH* expression, promoting *CD155* production in CRC cells. Moreover, *BCL9* was linked to adenomatous polyposis coli (APC) mutation involved in patient survival following anti-PD-L1 treatment. Ultimately, this study proved that *BCL9* inhibition altered cellular diversity within the tumor immune milieu and shed light on the role of *BCL9* in regulating *CD226* and *CD96* checkpoints. Using identical mice xenograft models, a group of scientists from China described that *BCL9* inhibition attenuated CRC growth *via* inhibiting TAM polarization from M0 to M2 phenotype that interfered with inflammatory actions of M0 and M1 TAMs. Based on the cellular landscape and transcription differences of TAMs after *BCL9* suppression, they demonstrated that regulation of Wnt signalling *via BCL9* suppression was expected to impair TAM-induced inflammation, CRC progression and immune surveillance (60). In a nutshell, unlike traditional immunotherapy, which targets solely CRC patients in late stages with MSI-H, scRNA-seq immune transcriptional studies offer an alternative option for precision medicine by targeting the Wnt signalling pathway *via BCL9* depletion.

## Immune transcriptomics identifies novel biomarker and revises immunotherapy strategies

The expanding pool of knowledge regarding the immunological complexity of the tumor microenvironment (TME) resulted in the discovery of a vast majority of previously unappreciated biomarkers, which played crucial roles in the modulation of CRC TIME. These biomarkers provide candidates for immunotherapy prediction, resulting in a paradigm shift in personalized immuno-oncology. The identification of new regulatory roles in neighboring immune cells *via* cancer biomarkers/gene expressions are not limited to T cells or TAMs as previously described. One such example is tumor specific innate lymphoid cells (ILCs). Single cell characterization of ILCs in healthy and CRC conditions was performed, with ILC1s, ILC3s, and ILC3/NKs subsets identified in the healthy gut; and ILC1-like and ILC2s subsets found to be tumor specific. Moreover, *SLAMF1* expression in ILCs had been discovered as an anti-tumor biomarker in CRC (68).

Other biomarkers such as tumor mutational burden (TMB), inflammation, alteration in a specific gene or signalling pathway (e.g. *BCL9* in Wnt pathway), APC mutation and MSI, allow stratification of patients for targeted immunotherapy and are consistent with several studies whereby TMB contributed to CRC immune landscape modelling (37); and *BCL9* depletion in Wnt signalling reprogrammed TIME, promoting anti-tumoral immune response (60, 67, 69). On the other hand, the conversion of TIME into a functionally inflamed immune hub *via* IL12 and Entinostat combinational therapy promoted and sustained the clinical benefits of immune therapy to a wider proportion of CRC patients. This study provides a rationale for combination therapy in the clinical setting for tailored immunotherapy (64). Furthermore, Wu et al. pinpointed the presence of energetic *MRC1* ^+^ *CCL18* ^+^ M2-like macrophages under an immunosuppressive state and susceptible to neoadjuvant chemotherapy (NAC) that restored anti-tumoral immune balance in TIME, suggesting the possibility of personalized immunotherapy in combination with NAC (65). In summary, the remodelling of an immune translational landscape in CRC appears promising in terms of the development of personalized cancer treatment and improvement in the selection of patients who may benefit from immunotherapy. Despite ground-breaking discoveries in single cell immune transcriptional studies, a comprehensive exploration of infiltrating immune cells in CRC TIME is still inadequate for clinical applications. **Table 1**.

**Table 1:** The summary of key discoveries on the single-cell transcriptional CRC immune landscape.

Type of Sample	Cell Infiltration	Key Finding	Number and Type of Cell	Sequencing Technology	Cells Identification/Screening	Reference
• Adjacent normal mucosa and CRC tissues	*FAP* ^+^ fibroblasts *SPP1* ^+^ macrophages	Identification of positive correlation between tumor specific *FAP* ^+^ fibroblasts and *SPP1* ^+^ macrophages Possible regulation of the interaction between *FAP* ^+^ fibroblasts and *SPP1* ^+^ macrophages *via* chemerin, TGF-β, and interleukin-1, and stimulation of the formation of immune-excluded desmoplastic structure and limitation of T cell infiltration Identification of patients with high *FAP* or *SPP1* expression achieved less therapeutic benefit from an anti-PD-L1 therapy cohort Potential therapeutic strategy *via* disrupting *FAP* ^+^ fibroblasts and *SPP1* ^+^ macrophages interaction to improve immunotherapy	• 54,103 single cells (29,481 adjacent non-malignant cells and 24,622 tumor cells)	Chromium system- 10X Genomics scRNA-seq Spatial transcriptomics (tumor tissue sections from four CRC patients)	Nine main cells: Endothelial cells B cells Epithelial cells Myeloid cells Plasma B cells Glial cells Mast cells Mesenchymal stem cells T/innate lymphoid cells (ILCs)	(63)
• Colorectal cancer liver metastasis (CRLM)	*MRC1* ^+^ *CCL18* ^+^ macrophages *SPP1* ^+^ macrophages *FOXP3* ^+^ regulatory T (Treg) cells	Extensive spatiotemporal reprogramming of metastatic immune microenvironment in CRLM *via CD45* ^+^ cell dynamics quantification Strong enrichment of immunosuppressive *MRC1* ^+^ *CCL18* ^+^ M2-like macrophages with a terminally differentiated state and metabolically energetic phenotype Reprogramming of cellular states of macrophages in neoadjuvant chemotherapy (NAC)-responsive patients, pinpointing the functional impacts of NAC on TIME	79,703 single cells from 13 treatment-naïve patients 36,284 single cells from 5 NAC-progressive disease (PD)/stable disease (SD) patients 62,643 cells from 6 NAC-partial response (PR) patients	Chromium system- 10X Genomics scRNA-seq (11 treatment naïve, 5 NAC-PD/SD, and 4 NAC-PR patients) Spatial transcriptomics (2 treatment naïve and 2 NAC-PR patients)	Seven main immune cells: Myeloid cells *CD8* ^+^ T cells *CD4* ^+^ T cells Natural killer (NK) cells Mucosal associated invariant T (MAIT) cells Neutrophils Treg cells	(65)
• Primary human CRC tumor tissues • Colorectal cancer hepatic metastases	*IgG* ^+^ plasma cells (infiltration level gradually decreased along the centre to the periphery of the tumor) Mast cells (enriched in stage III and IV CRC) T lymphocytes (cycling T cells enriched in primary CRC tumors)	Identification of B cells from early CRC tumor to be pre-B like expressing tumor suppressors, and the full development of plasma cells from B cells in advanced CRC tumors Supremacy of B and myeloid cells in immunoregulatory functions in CRC over *CD4* ^+^ Treg cells Discovery of the interplay between T, B and myeloid cells: a) B-cell/*CD52* ^+^ myeloid-cell interaction - Inhibition of *SIGLEC10* ^+^ T cells activation b) *CCL8* ^+^ cycling B-cell/*CCR5* ^+^ T-cell and *IgA* ^+^ *IGLC2* ^+^ plasma-cell/*CCR5* ^+^ T-cell interactions -Recruitment of *CCR5* ^+^ T-cell to tumor lesions	15,115 single immune and nonimmune cells from 18 CRC patients’ primary CRC tumors and hepatic metastases	Two scRNA-seq methods: Smart-seq2 (5,345 *CD45* ^+^ single cells) DNBelab C4 (9,770 single cells)	Four immune cell types: T cells B cells Myeloid cells Mast cells Three nonimmune cell types: Epithelial cells Endothelial cells Fibroblasts	(57)
• CRC specific innate lymphoid cells (ILC)	CRC tissue-specific ILC2s (ctILC2s)	Discovery of unique transcriptomic features of blood and tumor ILCs from CRC patients *via* single-cell immune landscape on ctILCs Uncovering an ILC1-like subpopulation unique to the tumor tissue from CRC patients Identification of ILC2s SLAMF1 as an anti-tumor biomarker in CRC	58,000 single total purified helper-like ILCs from blood samples from CRC patients, healthy blood, normal mucosa, and CRC tissue samples	Chromium system-10X Genomics scRNA-seq	Three ILC subsets in the healthy gut: ctILC1s subsets ctILC3s subsets ctILC3/NK subsets Two ILC subsets in CRC patients: Helper-like ILC1 subsets ILC2 subsets	(68)
• CRC adjacent normal cells • Precancerous CRC cells • CRC cells of different stages (stage I to IV)	T lymphocytes (including MAIT, *CTLA4* ^+^ and *CTLA4* ^-^ Treg cells)	Discovery of attenuated B-cell antigen presentation, distinct regulatory T-cell clusters with different origins and novel polyfunctional tumor related *SPP1* ^+^ tumor-associated macrophages (TAMs) Identification of increased *XCL1* ^+^ in T-cell clusters (*CD8* ^+^ cytotoxic T lymphocytes and exhausted *CD8* ^+^ T cells) linked with high status of tumor mutations Contribution of tumor mutational burden (TMB) in shaping CRC immune landscape Study of molecular mechanisms/epigenetic shaping of the tumor immune microenvironment *via* single-cell assay for transposase accessible chromatin using sequencing (scATAC-seq)	15,851 single cells from CRC adjacent normal tissue 8,299 single cells from precancerous CRC tissue 9,887 single cells from CRC tumor	Chromium system-10X Genomics scRNA-seq scATAC-seq (6,526 cells)	Seven clusters from total CRC cells: T cells B cells Myeloid cells MAIT cells NK cells Epithelial cells Fibroblasts	(37)
• Spleen and grown xenograft tumor from mouse models *via* EMT6 breast cancer and CT26 CRC cell lines	*CD4* ^+^ *FOXP3* ^+^ Treg cells CD8^+^ EMT6 T cells M1-like TAMs M2-like TAMs	Conversion of TIME into functionally inflamed immune hub *via* the concerted action of highly functional *CD8* ^+^ EMT6 T cells and activated neutrophils drive M1-like TAM polarization, leading to complete tumor eradication Facilitation of Entinostat in the accumulation of the necrosis-targeted recombinant murine immune-cytokine, NHS-rmIL12, in experimental mouse colon carcinomas and poorly immunogenic breast tumors	1,314 *CD45* ^+^ single cells from tumors treated with PBS 2,463 *CD45* ^+^ single cells from tumors treated with Entinostat 1,401 *CD45* ^+^ single cells from tumors treated with NHS-rmIL12 4,743 *CD45* ^+^ single cells from tumors treated with combination therapy	Chromium system-10X Genomics scRNA-seq	14 clusters from EMT6 tumor infiltrating *CD45* ^+^ cells: Eight TAM clusters One M2-like cluster Five undefined TAM clusters	(64)
• *BCL*9^+^/*BCL*9^-^ CT26 tumor from mouse models	• TAMs	Depletion of *BCL9*, resulting in TAM polarization inhibition from M0 to M2 and alteration of the CRC TIME, interfering with the inflammation of M0 and M1 *via* the Wnt signaling pathway	Single cells from 12 mice tumor samples (treated by hsBCL9_CT-_24, pGIPZ (inducible with doxycycline)-based lentiviral shRNAs and non-targeting shRNAs as a vehicle)	Chromium system-10X Genomics scRNA-seq	Six main cell clusters: Tumour cells Tumour-associated monocyte TAMs Tumour-associated endothelial cells T cells Fibroblast cells	(60)
• *BCL*9^-^ CT26 tumor from mouse models	Cells interacting with infiltrated leucocytes: Cancer-associated fibroblasts	Discovery of a pro-tumor effect of CAFs due to *BCL9* depletion Inhibition of abnormal activation of Wnt/β-catenin signal through BCL9 depletion benefits T-cell–mediated antitumor immune responses	Single cells from 6 CT26 mouse tumors treated with hsBCL9_CT-_24 or by a vehicle as control	Chromium system-10X Genomics scRNA-seq	Seven cell clusters from C0 to C6 according to their total cell numbers	(67)
• CT26 xenograft tumor from mouse models	CD8^+^ T cells Treg cells	Key roles of CD155-CD226 and CD155-CD96 checkpoints in cancer cell/*CD8* ^+^ T cell interaction Phosphorylation of VAV1 in CD8^+^ T cells *via* BCL9 suppression, mediating Wnt transcription Upregulation of *GLI1* and *PATCH* expression in promoting *CD155* expression in cancer cells.	Single cells from 8 CT26 mouse tumors treated with NT-shRNA, Bcl9-shRNA, hsBCL9_CT-_24 or by a vehicle as control	Chromium system-10X Genomics scRNA-seq	Six main cell clusters: CD8^+^ T cells Natural killer (NK) and T cells Treg cells Activated T cells Proliferation of T cells T helper cells	(69)
• Tumor and non-malignant human colon tissues	Three cells related to CRC tumor infiltration: Treg cells Myofibroblasts Myeloid cells	Correspondence of intercellular network reconstruction with the association between cancer cell signatures and specific stromal or immune cell populations Formation of immunosuppressive microenvironments controlled by Treg cells, myofibroblasts, and myeloid cells due to a genetic alteration in cancer cells Discovery of potential key marker genes such as CA2, PLAC8 and TSPAN1 to predict prognosis and immunotherapy response according to single cell expression atlas (SCEA) (70)	91,103 unsorted single cells from 23 Korean and 6 Belgian CRC patients	Chromium system-10X Genomics scRNA-seq	Six main cell clusters: Epithelial cells Stromal cells Myeloid cells T cells B cells Mast cells	(56)
• Primary human CRC tumor tissues	*CD103* ^+^ *CD69* ^+^ tumor-resident cytotoxic T cells T helper cells Memory *CD8* ^+/^γδ T cells	Enrichment of cytotoxic *Lin^–^CD7* ^+^ *CD127* ^–^ *CD56* ^+^ *CD45RO* ^+^ ILCs in mismatch-repair (MMR) deficit CRC tissues with tissue-resident (*CD103* ^+^ *CD69* ^+^) phenotypes Correlation of ILC with the infiltration of tumor-resident cytotoxic, helper and γδ T cells with highly similar activated (*HLA* ^-^ *DR* ^+^ *CD38* ^+^ *PD-1* ^+^) phenotypes Enrichment of *PD-1* ^+^ γδ T cells in MMR-deficient cancers and potential treatment *via* PD-1 checkpoint blockade	795 single CD45^+^ cells from seven CRC patients (four MMR-deficient and three MMR-proficient)	Chromium system-10X Genomics scRNA-seq	Seven main cell clusters: B cells Memory *CD4* ^+^ T cells Naive *CD4* ^+^ T cells Naive *CD8* ^+^ T cells Memory *CD8* ^+/^γδ T cells ILCs Myeloid cells	(55)
• Immune and stromal populations from CRC patients	*C1QC* ^+^ TAMs *SSPP1* ^+^ TAMs	Identification of specific macrophage and conventional dendritic cell (cDC) subsets as key mediators of cellular crosstalk in the TIME Discovery of conserved myeloid subsets in human and murine CRC: a) Two distinct TAM subsets with inflammatory and angiogenic signatures b) Two distinct TAM subsets with differential sensitivity to CSF1R blockade Activation of specific cDC1s; expansion of Th1-like and *CD8* ^+^ memory T cells *via* anti-CD40	Not stated but it is between 7,000 and 10,000 single cells	Chromium system-10X Genomics scRNA-seq Smart-Seq2 protocol	13 myeloid subsets: TPSAB1^+^ Mast cells *LILRA4* ^+^ plasmacytoid dendritic cells (DC) *CD1C* ^+^ cDC2 cells *BATF3* ^+^ cDC1 cells *CD14* ^+^ monocytes *CD16* ^+^ monocytes *CD14* ^+^ *CD16* ^+^ monocytes *NLRP3* ^+^ monocytes *PLTP* ^+^ monocytes *IL1B* ^+^ monocytes *FCN1* ^+^ monocyte-like cells *C1QC* ^+^ TAMs *SSPP1* ^+^ TAMs	(49)
• Primary human lung or CRC tumors and non-neoplastic counterpart	Treg cells	Enrichment of highly suppressive tumor-infiltrating Treg cells with elevations in many immunological checkpoints, and highly expressed unique characteristic markers on the cell surface, including interleukin-1 receptor 2 (*IL1R2*), programmed death (PD)-1 Ligand 1, PD-1 Ligand 2, and *CCR8* chemokine Association of high expression of Treg cell signature genes (LAYN, MAGEH1, or CCR8) in whole-tumor samples with a poor prognosis	858 single Treg cells (320 from CRC and 286 from NSCLC; 252 from PBMCs of healthy individuals)	C1 System -Fluidigm scRNA-seq	Three main subsets from primary human lung or CRC tumors and non-neoplastic counterparts: *CD4* ^+^ Treg cells *CD4* ^+^ Th1 cells *CD4* ^+^ Th17 cells	(53)
• Primary human CRC tumor tissues	Treg cells	Independent connection between *CD8* ^+^ effector, ‘exhausted’ T cells and tumor-resident *CD8* ^+^ effector memory cells, implicating a TCR-based fate decision Clonal exclusivity in most tumor infiltrating *CD4* ^+^ Treg cells and certain developmental linkage to several T helper cell clones Identification of two *IFNG* ^+^ TH1-like cell clusters in tumors that were associated with distinct IFNγ-regulating transcription factors —the *GZMK* ^+^ effector memory T cells, which were associated with EOMES and RUNX3, and *CXCL13* ^+^ *BHLHE40* ^+^ TH1-like cell clusters, which were associated with *BHLHE40* Preferential enrichment of *CXCL13* ^+^ *BHLHE40* ^+^ in patients with microsatellite-instable tumors, and favourable responses to immune-checkpoint blockade High expression of *IGGLR1* in both *CXCL13* ^+^ *BHLHE40* ^+^ TH1-like cells and *CD8* ^+^ exhausted T cells with co-stimulatory functions	11,138 single T cells from 4 MSI and 8 MSS CRC patients	FACS single sorting, followed Smart-Seq2 protocol	Eight *CD8* ^+^ and 12 *CD4* ^+^ T cell clusters	(54)

## Conclusion and future direction

Although conventional cancer transcriptomics focused on TME holds promise in cancer treatment, the treatment of advanced metastatic CRCs remains challenging. Hence, it is of paramount importance to explore innovative therapeutic targets/strategies. The transition towards single cell immune transcriptomics unveils the precise landscape of both immune and non-immune cells throughout CRC TIME. This immune-based approach not only translates gene signatures into a collective landscape, but it also investigates cellular interactions between immune cells and highlights their potential values as novel CRC classification systems as well as immunotherapy targets for personalized cancer treatment. With the growing number of studies on single-cell transcriptional profiling of cancer-associated immune cells, we believe that integrating and comprehensively characterizing these data would deepen our understanding of the immunobiology of CRC TIME. The new insights provided into cancer biology and metastasis may allow new applications in precision medicine.

## Author contributions

N-SAM contributed the idea. N-SAM and FYFT prepared the manuscript. FYFT prepared the figure. L-HL provided critical insights and edited the manuscript. All authors contributed to the article and approved the submitted version.

## Funding

The related research is funded by the Ministry of Higher Education Higher Institution Centre of Excellence Grant JJ-2021-002. The APC was funded by Monash University Malaysia.

## Acknowledgments

The authors acknowledge the supports provided by the universities and Ministry of Higher Education Malaysia.

## Conflict of interest

The authors declare that the research was conducted in the absence of any commercial or financial relationships that could be construed as a potential conflict of interest.

## Publisher’s note

All claims expressed in this article are solely those of the authors and do not necessarily represent those of their affiliated organizations, or those of the publisher, the editors and the reviewers. Any product that may be evaluated in this article, or claim that may be made by its manufacturer, is not guaranteed or endorsed by the publisher.
